# Effects of low concentrations of erythromycin, penicillin, and virginiamycin on bacterial resistance development *in vitro*

**DOI:** 10.1038/s41598-017-09593-4

**Published:** 2017-09-08

**Authors:** Beilei Ge, Kelly J. Domesle, Qianru Yang, Shenia R. Young, Crystal L. Rice-Trujillo, Sonya M. Bodeis Jones, Stuart A. Gaines, Marla W. Keller, Xin Li, Silvia A. Piñeiro, Brooke M. Whitney, Heather C. Harbottle, Jeffrey M. Gilbert

**Affiliations:** 1 U.S. Food and Drug Administration, Center for Veterinary Medicine, Office of Research, Division of Animal and Food Microbiology, Laurel, Maryland 20708 USA; 2 U.S. Food and Drug Administration, Center for Veterinary Medicine, Office of Surveillance and Compliance, Division of Animal Feeds, Rockville, Maryland 20855 USA; 3 U.S. Food and Drug Administration, Center for Veterinary Medicine, Office of New Animal Drug Evaluation, Division of Human Food Safety, Rockville, Maryland 20855 USA

## Abstract

Distillers grains are co-products of the corn ethanol industry widely used in animal feed. We examined the effects of erythromycin, penicillin, and virginiamycin at low concentrations reflective of those detected in distillers grains on bacterial resistance selection. At 0.1 µg/ml erythromycin, macrolide-resistant mutants were induced in one *Campylobacter coli* and one *Enterococcus faecium* strain, while these strains plus three additional *C*. *coli*, one additional *E*. *faecium*, and one *C*. *jejuni* also developed resistance when exposed to 0.25 µg/ml erythromycin. At 0.5 µg/ml erythromycin, a total of eight strains (four *Campylobacter* and four *Enterococcus*) obtained macrolide-resistant mutants, including two strains from each genus that were not selected at lower erythromycin concentrations. For penicillin, three of five *E*. *faecium* strains but none of five *Enterococcus faecalis* strains consistently developed resistance at all three selection concentrations. Virginiamycin at two M_1_:S_1_ ratios did not induce resistance development in four out of five *E*. *faecium* strains; however, increased resistance was observed in the fifth one under 0.25 and 0.5 µg/ml virginiamycin selections. Although not yet tested *in vivo*, these findings suggest a potential risk of stimulating bacterial resistance development in the animal gut when distillers grains containing certain antibiotic residues are used in animal feed.

## Introduction

Distillers grains, co-products of the corn ethanol industry, are widely used animal feed ingredients owing to their abundance and nutritional content^1^. Over the past 15 years, the expansion of the U.S. domestic ethanol industry has led to an exponential growth in distillers grains feed production^2^. In 2016, an estimated 42 million metric tons were produced, which were fed, in up to 40% of the ration^1^, to beef cattle (44%), dairy cattle (30%), swine (16%), poultry (9%), and others (1%)^2^. Roughly 27% of the total production was exported^2^.

For at least two decades, antibiotics such as virginiamycin and penicillin have been used to control bacterial contamination of commercial ethanol fermentations^1^. The steady increase in distillers grains feed production raised growing concerns over this practice^3^. In two nationwide surveys conducted by the U.S. Food and Drug Administration’s Center for Veterinary Medicine (FDA/CVM), several antibiotics, including virginiamycin, erythromycin, penicillin, and tylosin, have been detected at low concentrations (0.1 to 1.5 ppm) in some of the distillers grains products analyzed^4, 5^. There is concern that through the feeding of distillers grains, food-producing animals are exposed to these antibiotic residues on a continuous basis, which may give rise to antibiotic-resistant bacteria that could be passed down the food chain^3^.

Antibiotic resistance is a growing public health threat worldwide^6^. In the United States, an estimated 2 million illnesses and 23,000 deaths are caused by antibiotic-resistant bacteria each year^7^. Urgent and multifaceted efforts are therefore needed to curb resistance development in both the human clinical setting and in food production^8^. Traditionally, selection of resistant mutants occurs at antibiotic concentrations between the minimal inhibitory concentration (MIC) of a susceptible wild-type bacterial population (MIC_susc_) and that of a resistant one (MIC_res_), i.e., within the mutant selection window^9, 10^. The effect of lower concentrations of antibiotics such as those detected in distillers grains, at between one-tenth and one-half of the MIC_susc_ or lower, on the selection and enrichment of antibiotic-resistant bacteria is not well understood.

To gain insights into whether low concentrations of erythromycin, penicillin, and virginiamycin may select for resistant bacteria, a preliminary study was carried out at FDA/CVM, subjecting a small number of *Campylobacter* (tested for erythromycin only) and *Enterococcus* strains to limited antibiotic selection concentrations and exposure times^11^. *Campylobacter* is a leading cause of foodborne illnesses in the United States, and frequently colonizes the intestinal tracts of poultry, swine, and other food-producing animals^12^. The opportunistic *Enterococcus* is also highly prevalent in the gut of food-producing animals as well as humans, and is a leading cause of nosocomial infections^13^. The preliminary study showed that penicillin at 1 µg/ml, virginiamycin at 0.1 and 1 µg/ml, a penicillin/virginiamycin blend at 1/0.075 µg/ml, and erythromycin at 0.1 µg/ml did not select for resistant *Enterococcus* phenotypes. However, erythromycin at both 0.25 and 0.5 µg/ml stimulated resistance development in *Enterococcus*. In *Campylobacter*, 0.5 µg/ml erythromycin (the only concentration tested) did not select for resistant mutants^11^. Considering the importance of these antibiotics in human and veterinary medicine, these findings support the need for further investigation into the microbiological impact of erythromycin and other residues detected in distillers grains^14^.

The present study aimed to comprehensively examine the effects of three low concentrations (0.1, 0.25, and 0.5 µg/ml) of erythromycin, penicillin, and virginiamycin (a mixture of two components, virginiamycin M_1_ and virginiamycin S_1_) on the development of antibiotic-resistant bacteria. Multiple *Campylobacter* (tested for erythromycin only) and *Enterococcus* strains were used as sentinel organisms for Gram-negative and Gram-positive bacteria, respectively.

## Results

### Resistance selection at low concentrations of erythromycin

The erythromycin MICs of twenty parent strains ranged from 0.25 to 1 µg/ml for *Campylobacter* and ≤0.25 to 1 µg/ml for *Enterococcus* (Table 1). In two independent trials, resistance development was stimulated in some strains of both bacterial genera during culture passages at all three erythromycin selection concentrations, with mutant MICs ranging from 64 to >2048 µg/ml for *Campylobacter* and 8 to >2048 µg/ml for *Enterococcus* (Table 2). At 0.1 µg/ml erythromycin, resistant mutants were observed in *Campylobacter coli* N20290 and *Enterococcus faecium* N39268 only. The same two strains, plus three additional *C*. *coli*, one additional *E*. *faecium*, and one *C*. *jejuni* strain also developed resistance when exposed to 0.25 µg/ml erythromycin. At 0.5 µg/ml erythromycin, eight strains (three *C*. *coli*, one *C*. *jejuni*, and two each *E*. *faecalis* and *E*. *faecium*) obtained resistant mutants, including two from each genus (one *C*. *coli*, one *C*. *jejuni*, and two *E*. *faecalis*) that were not selected at 0.1 and 0.25 µg/ml erythromycin. Notably, *E*. *faecium* N39268 mutants were selected at all three erythromycin concentrations, whereas no mutants were selected for three *C*. *jejuni*, three *E*. *faecalis*, two *E*. *faecium*, and the single *E*. *hirae* strain. All *Campylobacter* and *E*. *faecalis* mutations occurred at passages 2 or 3. Many mutants were generated outside the mutant selection window, i.e., at erythromycin concentrations lower than parent MICs, therefore with selection ratios (defined as the ratio between antibiotic selection concentration and parent strain MIC, i.e., MIC_susc._) less than 1 (Table 2).

**Table 1:** Characteristics of *Campylobacter* and *Enterococcus* strains used in this study.

Genus and species	Strain^a–c^	Antibiotic tested	Source	MIC (µg/ml)		
				Erythromycin	Penicillin	Quinupristin/Dalfopristin
*Campylobacter coli* (*n* = 5)	N15947^a^	Erythromycin only	Pork chop	0.5	N/A	N/A
	N16008^a^	Erythromycin only	Ground beef	1	N/A	N/A
	N20290^a^	Erythromycin only	Chicken breast	0.25	N/A	N/A
	N20293^a^	Erythromycin only	Chicken breast	0.5	N/A	N/A
	N40971^a^	Erythromycin only	Ground turkey	1	N/A	N/A
*Campylobacter jejuni* (*n* = 5)	N9328	Erythromycin only	Pork chop	0.5	N/A	N/A
	N16006^a^	Erythromycin only	Ground beef	0.25	N/A	N/A
	N20289^a^	Erythromycin only	Chicken breast	0.5	N/A	N/A
	N20292	Erythromycin only	Chicken breast	0.25	N/A	N/A
	N39676	Erythromycin only	Ground turkey	0.25	N/A	N/A
*Enterococcus faecalis* (*n* = 7)	N17045^a^	Erythromycin, penicillin	Ground beef	1	2	4
	N39253	Erythromycin only	Ground turkey	1	4	8
	N39282	Erythromycin only	Pork chop	0.5	4	8
	N39331	Erythromycin, penicillin	Chicken breast	1	4	8
	N39462^a^	Erythromycin, penicillin	Ground beef	≤0.25	4	16
	N40185	Penicillin only	Pork chop	0.5	1	8
	N40682	Penicillin only	Ground turkey	>8	1	16
*Enterococcus faecium* (*n* = 7)	N17044^a^	Erythromycin only	Ground turkey	1	>16	16
	N39268^a^	Erythromycin, penicillin, virginiamycin	Ground beef	1	2	1
	N39411^b^	Penicillin, virginiamycin	Ground turkey	4	1	2
	N39482^b^	Penicillin, virginiamycin	Chicken breast	≤0.25	≤0.25	≤0.5
	N39577^b^	Penicillin, virginiamycin	Pork chop	1	0.5	1
	N41264	Erythromycin only	Pork chop	0.5	1	2
	N42162^c^	Erythromycin, penicillin, virginiamycin	Ground beef	≤0.25	4	4
*Enterococcus hirae* (*n* = 1)	N17030S	Erythromycin only	Chicken breast	≤0.25	1	2

^a–c^Strains that developed resistance at low concentrations (0.1, 0.25, and 0.5 µg/ml) of erythromycin (a), penicillin (b), and virginiamycin (c), respectively.

**Table 2:** Selection of resistant *Campylobacter* and *Enterococcus* mutants at low concentrations of erythromycin (ERY).

ERY conc. (µg/ml)	Genus and species	Strain^a,b^	Source	Confirmed mutants occurred at passages^c^		ERY MIC (µg/ml)^d,e^		No. of confirmed mutants
				Trial 1	Trial 2	Parent	Mutants	
0.1	*C*. *coli*	N20290^a^	Chicken	P3	P2-P3	0.25^d^	256->2048	12
	*E*. *faecium*	N39268^b^	Beef	P1-P3	P1, P3	1^d^	8–64	13
0.25	*C*. *coli*	N15947^a^	Pork	None	P3	0.5^d^	64	1
		N20290^a^	Chicken	P3	P2	0.25	256–1024	6
		N20293^a^	Chicken	None	P2-P3	0.5^d^	512–2048	6
		N40971	Turkey	P2-P3	P2	1^d^	64–1024	8
	*C*. *jejuni*	N20289	Chicken	None	P3	0.5^d^	1024	1
	*E*. *faecium*	N17044^a^	Turkey	P1-P3	P1-P3	1^d^	8–32	20
		N39268^b^	Beef	P1-P3	P1-P3	1^d^	8–64	16
0.5	*C*. *coli*	N15947^a^	Pork	P2-P3	P2-P3	0.5	64–2048	13
		N16008	Beef	P3	None	1^d^	64–128	4
		N20293^a^	Chicken	P3	P3	0.5	1024–2048	6
	*C*. *jejuni*	N16006	Beef	P2-P3	None	0.25	512–1024	7
	*E*. *faecalis*	N17045	Beef	P3	None	1^d^	1024	2
		N39462	Beef	P2-P3	P2-P3	≤0.25	≥2048	16
	*E*. *faecium*	N17044^a^	Turkey	P1-P3	P1-P3	1^d^	8–16	22
		N39268^b^	Beef	P1-P3	P1-P3	1^d^	8–64	12

The following strains did not obtain resistant mutants at any of the three ERY concentrations: *C*. *jejuni* N9328, N20292, and N39676; *E*. *faecalis* N39253, N39282, and N39331; *E*. *faecium* N41264; and *E*. *hirae* N17030S.

^a^Resistant mutants were selected at two ERY concentrations.

^b^Resistant mutants were selected at three ERY concentrations.

^c^P1, P2, and P3 stand for passages 1, 2, and 3, respectively.

^d^Resistance selection occurred outside the mutant selection window, i.e., the ERY concentrations used for selection were lower than the parent MICs for ERY.

^e^ERY resistant breakpoints of ≥32 µg/ml and ≥8 µg/ml were used for *Campylobacter* spp. and *Enterococcus* spp., respectively.

Regardless of the erythromycin concentration, selection ratio, and number of passages, *Campylobacter* strains overall had a higher incidence (70%) of resistance development than *Enterococcus* (40%) (*P* > 0.05). The selective effect was more pronounced among *C*. *coli* (100%) than *C*. *jejuni* (40%) (*P* < 0.05) and among *E*. *faecium* (50%) than *E*. *faecalis* (40%) (*P* > 0.05). Erythromycin at 0.25 and 0.5 µg/ml induced resistance in significantly larger percentages of *Campylobacter* and *Enterococcus* strains than those at 0.1 µg/ml (*P* < 0.05). Notably, resistant mutants of *C*. *jejuni* were only obtained at 0.25 and 0.5 µg/ml erythromycin, whereas *E*. *faecalis* mutants were only obtained at 0.5 µg/ml erythromycin (Table 3). The selection ratio also played a major role in the process with higher ratios generally associated with stronger selective effects. A selection ratio of 1/4 had the highest frequencies of mutated strains in both *Campylobacter* and *Enterococcus* (data not shown). Passages 2 and 3 were linked to stronger selection effect than passage 1, which was statistically significant in *Campylobacter* (*P* < 0.05) (data not shown).

**Table 3:** Antibiotic resistance profiles of *Campylobacter* and *Enterococcus* parent and mutant strains selected at low concentrations of erythromycin (ERY).

Genus and species	Strain	Resistance profile (No. of antibiotic classes resistant to)^a^		ERY conc. (µg/ml)	No. of mutants
		Parent	Mutants		
*C*. *coli*	N15947	TET (1)	AZI-CLI-ERY-TEL-TET (4)	0.5	1
			AZI-CLI-ERY-TEL(I)-TET (3)	0.5	1
			AZI-ERY-TEL-TET (3)	0.5	4
			ERY-TEL-TET (3)	0.25, 0.5	6
			AZI-ERY-TEL(I)-TET (2)	0.5	1
			ERY-TEL(I)-TET (2)	0.5	1
	N16008	TET (1)	ERY-TEL-TET (3)	0.5	4
	N20290	None	AZI-CLI-ERY-TEL (3)	0.1	2
			AZI-CLI-ERY (2)	0.1	3
			AZI(I)-ERY-TEL (2)	0.1	1
			ERY-TEL (2)	0.1, 0.25	3
			AZI-ERY (1)	0.1	1
			AZI(I)-ERY-TEL(I) (1)	0.1	1
			ERY-TEL(I) (1)	0.1, 0.25	5
			ERY (1)	0.1, 0.25	2
	N20293	None	AZI-ERY-TEL (2)	0.25, 0.5	4
			ERY-TEL (2)	0.25, 0.5	8
	N40971	CIP-NAL-TET (2)	CIP-ERY-NAL-TEL-TET (4)	0.25	7
			CIP-ERY-NAL-TET (3)	0.25	1
*C*. *jejuni*	N16006	CIP-NAL (1)	AZI(I)-CIP-ERY-NAL-TEL (3)	0.5	3
			CIP-ERY-NAL-TEL(I) (2)	0.5	4
	N20289	None	ERY (1)	0.25	1
*E*. *faecalis*	N17045	ERY(I)-LIN-Q/D-TET (3)	CIP(I)-ERY-LIN-Q/D-TET (4)	0.5	2
	N39462	CIP(I)-LIN-Q/D (2)	CIP(I)-ERY-LIN-Q/D-TYL (3)	0.5	15
			CIP(I)-ERY-LIN-Q/D (3)	0.5	1
*E*. *faecium*	N17044	CIP-ERY(I)-LIN-NIT(I)-PEN-Q/D-TET (5)	CIP-DAP-ERY-LIN-NIT(I)-PEN-Q/D-TET-TGC (8)	0.5	1
			CIP-DAP-ERY-LIN-PEN-Q/D-TET-TGC (8)	0.25	1
			CIP-DAP-ERY-LIN-NIT(I)-PEN-Q/D-TET (7)	0.25, 0.5	10
			CIP-DAP-ERY-LIN-PEN-Q/D-TET (7)	0.25	3
			CIP-ERY-LIN-NIT(I)-PEN-Q/D-TET-TGC (7)	0.25, 0.5	3
			CIP-ERY-LIN-LZD(I)-NIT(I)-PEN-Q/D-TET (6)	0.5	1
			CIP-ERY-LIN-NIT(I)-PEN-Q/D-TET (6)	0.25, 0.5	14
			CIP-ERY-LIN-PEN-Q/D-TET (6)	0.25, 0.5	9
	N39268	ERY(I)-LIN-NIT(I)-TET (2)	CHL(I)-CIP(I)-DAP-ERY-LIN-NIT-TET (5)	0.25	1
			CHL(I)-CIP(I)-ERY-LIN-NIT-TET (4)	0.1, 0.25	3
			CHL(I)-CIP(I)-ERY-LIN-NIT(I)-TET (3)	0.1, 0.25, 0.5	19
			CHL(I)-CIP(I)-ERY-LIN-TET (3)	0.25, 0.5	3
			CHL(I)-ERY-LIN-NIT(I)-TET (3)	0.1, 0.25, 0.5	6
			CHL(I)-ERY-LIN-TET (3)	0.1, 0.25	2
			CIP(I)-ERY-LIN-NIT(I)-TET (3)	0.25	3
			ERY-LIN-NIT(I)-TET (3)	0.1, 0.5	4

^a^Antibiotics followed by I in parenthesis are intermediate. Underlined are resistance profiles present in mutants but absent in parents. For DAP and TGC, resistant breakpoints have not been established; non-susceptible mutants were reported.

Antibiotic abbreviations are: AZI, azithromycin; CHL, chloramphenicol; CIP, ciprofloxacin; CLI, clindamycin; DAP, daptomycin; ERY, erythromycin; LIN, lincomycin; LZD, linezolid; NAL, nalidixic acid; NIT, nitrofurantoin; PEN, penicillin; Q/D, quinupristin/dalfopristin; TEL, telithromycin; TET, tetracycline; TGC, tigecycline; and TYL, tylosin.

Besides erythromycin, some mutants also demonstrated resistance to other macrolides, such as azithromycin in three *C*. *coli* strains and tylosin in *E*. *faecalis* strain N39462 (Table 3). Co-resistance to telithromycin (a ketolide) and/or clindamycin (a lincosamide) was common among *Campylobacter* mutants, whereas some *E*. *faecium* mutants also showed resistance to nitrofurantoin (a nitrofuran) and non-susceptibility to daptomycin (a lipopeptide) and/or tigecycline (a glycylcycline). Intermediate resistance (MICs above intermediate breakpoint but below resistant breakpoint) to these antibiotics (azithromycin, clindamycin, nitrofurantoin, and telithromycin) was observed as well (Table 3).

### Resistance selection at low concentrations of penicillin

The penicillin MICs of ten *Enterococcus* parent strains ranged from 1 to 4 µg/ml for *E*. *faecalis* and ≤0.25 to 4 µg/ml for *E*. *faecium* (Table 1). In three independent trials, none of the five *E*. *faecalis* strains obtained penicillin-resistant mutants even after 15 passages, and neither did two out of five *E*. *faecium* strains. In direct contrast, *E*. *faecium* strains N39411, N39482, and N39577 consistently developed penicillin resistance at all three selection concentrations, with mutant MICs ≥ 16 µg/ml. Such difference in resistance selection between the two *Enterococcus* species was statistically significant (*P* < 0.05). Most mutants emerged between passages 7 and 10, and some as early as passage 1. Statistical analysis confirmed the link between higher numbers of passages and stronger resistance selection (*P* < 0.05). Similar to erythromycin, most penicillin mutants were generated outside the mutant selection window, i.e., at selection ratios less than 1 (Table 4). Interesting to note, the three *E*. *faecium* strains had parent MICs ranging from ≤0.25 to 1 µg/ml (selection ratios from 1/10 to ≥2) while the other two had parent MICs of 2 and 4 µg/ml (selection ratios from 1/40 to 1/4). This highlights the important role selection ratio played in the process with higher ratios generally associated with stronger selective effects (*P* < 0.05).

**Table 4:** Selection of resistant *Enterococcus* mutants at low concentrations of penicillin (PEN).

PEN conc. (µg/ml)	Strain^a^	Source	Confirmed mutants occurred at passages^b^			PEN MIC (µg/ml)^c,d^		No. of confirmed mutants
			Trial 1	Trial 2	Trial 3	Parent	Mutants	
0.1	N39411	Turkey	P7-P8, P10	P7-P10	P8-P9	1^c^	≥16	16
	N39482	Chicken	P8-P10	P8-P10	P1-P3	≤0.25^c^	≥16	18
	N39577	Pork	P3-P4, P7-P10	P7-P9	P2-P3, P7-P10	0.5^c^	≥16	35
0.25	N39411	Turkey	P8	P7-P10	P7-P9	1^c^	≥16	15
	N39482	Chicken	P8-P10	P2-P3, P7-P10	P3, P7-P10	≤0.25	≥16	25
	N39577	Pork	P2-P3, P7-P10	P7-P10	P7-P8, P10	0.5^c^	≥16	24
0.5	N39411	Turkey	P4, P7-P8, P10	P7-P10	P7-P8	1^c^	≥16	17
	N39482	Chicken	P1, P8-P10	P2-P3, P7-P8, P10	None	≤0.25	≥16	15
	N39577	Pork	P4, P7-P10	P2-P4, P7-P10	P2-P3, P7-P10	0.5	≥16	43

The following strains did not obtain resistant mutants at any of the three PEN concentrations: *E*. *faecalis* N17045, N39331, N39462, N40185, and N40682; and *E*. *faecium* N39268 and N42162.

^a^All three strains were *E*. *faecium*. ^b^P1 to P10 stand for passages 1 to 10, respectively. ^c^Resistance selection occurred outside the mutant selection window, i.e., the PEN concentrations used for selection were lower than the parent MICs for PEN. ^d^PEN resistant breakpoint of ≥16 µg/ml was used.

Besides penicillin, three *E*. *faecium* N39411 mutants also obtained resistance to erythromycin, to which the parent had an intermediate MIC (Table 5). Similarly, eight *E*. *faecium* N39577 mutants developed resistance to ciprofloxacin and/or erythromycin, to which the parent was intermediate. Non-susceptibility to daptomycin and/or tigecycline was observed in some *E*. *faecium* N39482 and N39577 mutants (Table 5). The MIC increases for the above mentioned antibiotics were 2–4 fold (data not shown).

**Table 5:** Antibiotic resistance profiles of *Enterococcus* parent and mutant strains selected at low concentrations of penicillin (PEN).

Genus and species	**Strain**	Resistance profile (No. of antibiotic classes resistant to)^a^		PEN con. (µg/ml)	No. of mutants
		Parent	Mutants		
*E*. *faecium*	N39411	ERY(I)-LIN-NIT(I)-Q/D(I) (1)	ERY-LIN-NIT(I)-PEN-Q/D(I) (3)	0.1, 0.25	3
			ERY(I)-LIN-NIT(I)-PEN-Q/D(I) (2)	0.1, 0.25, 0.5	44
			ERY(I)-LIN-LZD(I)-NIT(I)-PEN-Q/D(I) (2)	0.1	1
	N39482	CIP-NIT-TET (3)	CIP-DAP-NIT-PEN-TET (5)	0.1	1
			CIP-NIT-PEN-TET-TGC (5)	0.1	1
			CIP-NIT-PEN-TET (4)	0.1, 0.25, 0.5	56
	N39577	CIP(I)-ERY(I)-NIT(I) (None)	CIP-ERY-NIT(I)-PEN (3)	0.5	1
			CIP-ERY(I)-NIT(I)-PEN (2)	0.1, 0.5	7
			CIP(I)-DAP-ERY(I)-NIT(I)-PEN (2)	0.5	1
			CIP(I)-ERY(I)-LZD(I)-NIT(I)-PEN (1)	0.5	1
			CIP(I)-ERY(I)-NIT(I)-PEN (1)	0.1, 0.25, 0.5	91
			CIP(I)-ERY(I)-PEN (1)	0.25	1

^a^Antibiotics followed by I in parenthesis are intermediate. Underlined are resistance profiles present in mutants but absent in parents. For DAP and TGC, resistant breakpoints have not been established; non-susceptible mutants were reported.

Antibiotic abbreviations are: CIP, ciprofloxacin; DAP, daptomycin; ERY, erythromycin; LIN, lincomycin; LZD, linezolid; NIT, nitrofurantoin; PEN, penicillin; Q/D, quinupristin/dalfopristin; TET, tetracycline; and TGC, tigecycline.

### Resistance selection at low concentrations of virginiamycin

The Q/D MICs of five *E*. *faecium* strains used for virginiamycin selection ranged from ≤0.5 to 4 µg/ml (Table 1). In two independent trials, four strains did not develop resistance when exposed to any of the three concentrations of virginiamycin at either M_1_:S_1_ ratio (0.5:1 or 5:1). The fifth one, *E*. *faecium* N42162, was initially resistant to virginiamycin with Q/D MIC of 4 µg/ml. At 0.5 µg/ml virginiamycin in both trials and 0.25 µg/ml in one trial, *E*. *faecium* N42162 mutants with higher Q/D MICs (8–16 µg/ml) were obtained at both M_1_:S_1_ ratios. All mutants emerged after passage 7 and were selected outside the mutant selection window (Table 6).

**Table 6:** Selection of resistant *Enterococcus faecium* mutants at low concentrations of virginiamycin (VIR).

VIR conc. (µg/ml)	VIR ratio (M_1_:S_1_)	Strain	Source	Confirmed mutants occurred at passages^a^		Q/D MIC (µg/ml)^b,c^		No. of confirmed mutants
				Trial 1	Trial 2	Parent	Mutants	
0.25	0.5:1	N42162	Beef	P9-P10	None	4^b^	8–16	4
	5:1	N42162	Beef	P7	None	4^b^	4–8	2
0.5	0.5:1	N42162	Beef	P9-P10	P10	4^b^	4–16	6
	5:1	N42162	Beef	P9-P10	P8-P10	4^b^	4–16	9

The following strains did not obtain resistant mutants at any of the three VIR concentrations at two ratios: *E*. *faecium* N39268, N39411, N39482, and N39577.

*E*. *faecium* N42162 did not develop resistance at 0.1 µg/ml VIR at two ratios.

^a^P1 to P10 stand for passages 1 to 10, respectively.

^b^Resistance selection occurred outside the mutant selection window, i.e., the VIR concentrations used for selection were lower than the parent MICs for Q/D.

^c^Q/D, quinupristin/dalfopristin, was tested as a proxy for VIR and a resistant breakpoint of ≥4 µg/ml was used.

Besides higher Q/D MICs, some *E*. *faecium* N42162 mutants also obtained resistance to kanamycin, nitrofurantoin, and tylosin (Table 7), with MICs increasing by 4–8 fold compared to the parent strain (data not shown). It is noted that the parent strain had an intermediate MIC to nitrofurantoin.

**Table 7:** Antibiotic resistance profiles of *Enterococcus faecium* parent and mutant strains selected at low concentrations of virginiamycin (VIR).

VIR ratio (M_1_:S_1_)	Genus and species	**Strain**	Resistance profile (No. of antibiotic classes resistant to)^a^		VIR con. (µg/ml)	No. of mutants
			Parent	Mutants		
0.5:1	*E*. *faecium*	N42162	LIN-Q/D (2)	CIP(I)-ERY(I)-KAN-LIN-NIT-Q/D-TYL (5)	0.5	1
				CIP(I)-LIN-NIT(I)-Q/D-TYL (3)	0.25, 0.5	3
				CIP(I)-LIN-Q/D-TYL (3)	0.5	1
				LIN-NIT(I)-Q/D-TYL (3)	0.5	1
				LIN-Q/D-TYL (3)	0.5	1
				CIP(I)-LIN-LZD(I)-NIT(I)-Q/D (2)	0.25	1
				CIP(I)-LIN-NIT(I)-Q/D (2)	0.5	1
				LIN-NIT(I)-Q/D-TYL(I) (2)	0.25	1
5:1	*E*. *faecium*	N42162	LIN-Q/D (2)	CIP(I)-ERY(I)-KAN-LIN-NIT-Q/D-TYL (5)	0.5	2
				CIP(I)-ERY(I)-KAN-LIN-NIT(I)-Q/D-TYL (4)	0.5	1
				CIP(I)-ERY(I)-KAN-LIN-NIT(I)-Q/D-TYL(I) (3)	0.5	1
				CIP(I)-ERY(I)-LIN-NIT-Q/D-TYL(I) (3)	0.5	1
				CIP(I)-ERY(I)-LIN-NIT(I)-Q/D-TYL(I) (2)	0.5	1
				CIP(I)-LIN-NIT(I)-Q/D (2)	0.25	1
				LIN-NIT(I)-Q/D (2)	0.5	3
				LIN-Q/D (2)	0.25	1

^a^Antibiotics followed by I in parenthesis are intermediate. Underlined are resistance profiles present in mutants but absent in parents.

Antibiotic abbreviations are: CIP, ciprofloxacin; ERY, erythromycin; KAN, kanamycin; LIN, lincomycin; LZD, linezolid; NIT, nitrofurantoin; Q/D, quinupristin/dalfopristin; TIG, and TYL, tylosin.

## Discussion

Much of the literature on antibiotic resistance focuses on tolerance/adaptation to high dosages that typically occur in a clinical setting. The effects of sub-MIC, sub-inhibitory, sub-lethal, or sub-therapeutic concentrations of antibiotics on the evolution of resistant bacteria are just beginning to be understood^15–19^. One study showed that after exposing 20 independent lineages of *Escherichia coli* K-12 MG1655 and *Salmonella enterica* Typhimurium LT2 constantly to 1/10 MIC of ciprofloxacin and 1/4 MIC of streptomycin, respectively, almost all lineages contained resistant subpopulations with MICs several folds higher than the parents^17^. Another study reported the *de novo* acquisition of resistance to amoxicillin, enrofloxacin, and tetracycline by *E*. *coli* K-12 MG1655 in the presence of sub-lethal antibiotic concentrations^19^. Nonetheless, most of the studies were carried out in model organisms in molecular biology, such as a single laboratory strain of *E*. *coli*. To our knowledge, this is the first study where multiple wild-type *Campylobacter* and/or *Enterococcus* strains were used to evaluate the development of resistance against three antibiotics at the concentrations detected in distillers grains.

Our data demonstrate that low concentrations (0.1, 0.25, and 0.5 µg/ml) of erythromycin, penicillin, and virginiamycin can select for resistant *Campylobacter* and *Enterococcus* variants, and the selective effects differed among strains, species, and genera. Such discrepancies can be partially explained by the different genetic backgrounds of the strains, resistance characteristics at genus and species levels, types of mutations conferring resistance, and fitness costs (defined as reduced competitiveness in the absence of antibiotics) associated with the mutations^20^. Historically, the reported frequency of macrolide resistance in *C*. *coli* derived from both humans and food animals is much higher than that in *C*. *jejuni* ^21, 22^. Not surprisingly, a significantly higher percentage of *C*. *coli* (100%) developed macrolide resistance than *C*. *jejuni* (40%) in the present study (*P* < 0.05). Furthermore, one *C*. *coli* strain obtained resistant mutants even at the lowest erythromycin concentration tested, whereas *C*. *jejuni* mutants were obtained at 0.25 and 0.5 µg/ml erythromycin only. Our data also suggest that *E*. *faecium* had a stronger tendency than *E*. *faecalis* to develop erythromycin resistance because *E*. *faecium* mutants were selected at all three erythromycin concentrations while *E*. *faecalis* only at 0.5 µg/ml. For penicillin, the finding that three *E*. *faecium* strains consistently developed resistance agrees with species-specific resistance characteristics: *E*. *faecium* is inherently more resistant to β-lactam antibiotics than *E*. *faecalis* and now widespread, high-level resistance to ampicillin has been observed among clinical *E*. *faecium* isolates^23, 24^. The differential selection of Q/D-resistant *E*. *faecium* mutants by virginiamycin was largely attributable to strain-specific resistance traits, highlighting the importance of using multiple strains in antibiotic selection experiments.

Under our experimental design (a closed system without introducing other strains), gene mutation, either pre-existing or *de novo* selected, was most likely the single most important route contributing to resistance development, whereas horizontal gene transfer was unlikely to have played a role. Many mutations/genes have been reported that account for resistance to the three antibiotics tested. Macrolide resistance in *Campylobacter* is mainly associated with point mutations in domain V of the 23 S rRNA and/or ribosomal proteins L4 and L22, active efflux, and rRNA methylation^25, 26^. In *Enterococcus*, modification of the 23 S rRNA target confers co-resistance to macrolides, lincosamides, and streptogramin B (quinupristin or virginiamycin S_1_), known as the MLS_B_ phenotype^23, 27^. Drug inactivating enzymes and efflux also account for resistance to streptogramin antibiotics^23, 27^. Enterococcal resistance to β-lactams has been linked to mutations in the low-affinity penicillin binding protein *pbp5* gene and/or genes coding for other species-specific proteins involved in cell wall synthesis such as L,D-transpeptidases (Ldt_*fm*_), and β-lactamase^27, 28^. It is noteworthy that mutants generated in this study had MICs significantly higher (up to 8,192 fold above MIC_susc_) than the concentrations of antibiotics to which the parent strains were exposed (Tables 2 and 4). Genome-wide identification of resistance determinants among these mutants is currently under way. We anticipate detecting resistance mutations both specific to a particular drug and shared by multiple drugs, such as efflux pumps conferring resistance to multiple antibiotics, and may also include some potential new mechanisms^20, 29^, which may partly explain the co-resistance observed in some mutants.

One especially critical factor for resistance selection at sub-MIC concentrations of antibiotics is the variation in fitness cost incurred by different types of mutations^15, 17, 30^. Since susceptible bacteria are not killed at such low antibiotic concentrations but only grow slower, mutants will be competitive only when they carry fitness costs lower than the growth reduction in susceptible populations^15, 17^. A recent study reported fitness costs (measured by mutant growth rate reduction in comparison to the parent) of various resistance mutations in the range of 0.2 to 3% among *E*. *coli* and *S*. Typhimurium mutants selected by ciprofloxacin and streptomycin/tetracycline, respectively^16, 30^. Therefore, we expect resistant mutants obtained in the present study to carry variable degrees of low-fitness-cost mutations which accumulated and evolved over many generations through the selection process.

Aside from strain parameters, our data show that selective effects also differed by antibiotic selection concentrations and ratios, and number of passages (i.e., exposure time). The level of erythromycin, where no resistance selection occurred among 18 *Campylobacter* and *Enterococcus* strains was 0.1 µg/ml, which could be even lower for one *C*. *coli* and one *E*. *faecium* strain. The level of penicillin where no resistance selection occurred among most *Enterococcus* was greater than 0.5 µg/ml, while 0.1 µg/ml penicillin still selected for resistance in three *E*. *faecium* strains. For virginiamycin, the level where no resistance selection occurred was greater than 0.5 µg/ml for 4 *E*. *faecium* strains and 0.1 µg/ml for the fifth one. Taking into consideration the parent MICs (MIC_susc._), it is apparent that the selection ratio is a more appropriate parameter in this context. A similar term “minimal selective concentration (MSC)” was coined recently in two pioneering studies as the lowest antibiotic concentration that selects for a given resistance mutation in competition experiments^17, 18^. In essence, MSC measures the antibiotic concentration needed to overcome the fitness cost of each resistance determinant, i.e., for the mutant strain to be competitive over the susceptible population, and is expressed as a fraction of the MIC_susc_. The smaller the fitness cost is, the lower the anticipated MSC^15, 30^. Using slightly different approaches, the two studies reported the MSCs in *E*. *coli* for two antibiotics (1/5 of the MIC_susc._ for ciprofloxacin and 1/20 of the MIC_susc._ for tetracycline)^18^ and in *E*. *coli* and *S*. Typhimurium for three antibiotics (1/230 to 1/10 of the MIC_susc._ for ciprofloxacin in *E*. *coli* and 1/4 and 1/100 of the MIC_susc._ for streptomycin and tetracycline, respectively, in *S*. Typhimurium)^17^. In the present study, the lowest selection ratios among those tested that still generated resistant mutants were 1/4 in *Campylobacter* and 1/10 in *Enterococcus* for erythromycin (Table 2), and 1/10 and 1/16 in *E*. *faecium* for penicillin (Table 4) and virginiamycin (Table 6), respectively. Nonetheless, an optimum selection ratio of 1/4 was noted in erythromycin experiments for both *Campylobacter* and *Enterococcus* and penicillin selection showed a strong preference for strains with lower MIC_susc._ (i.e., higher selection ratio). This suggests that resistance selection at sub-MIC concentrations of antibiotics favors antibiotic concentrations not too distant from the MIC_susc._, although further studies are warranted to confirm this hypothesis.

The development and dissemination of antibiotic resistance in bacterial pathogens is of significant public health concern globally^6^. Drug-resistant *Campylobacter* and vancomycin-resistant *Enterococcus* are among current resistance threats in the U.S.^7^. As outlined in FDA’s Guidance for Industry #209, the use of medically important antibiotics, including macrolides, β-lactams, and streptogramins, at sub-therapeutic levels in food animals poses a human health risk due to the potential for resistance development^31^. The concentrations of antibiotics tested in this study are reflective of those detected in distillers grains products in two nationwide surveys conducted by the FDA/CVM^4, 5^. Another survey reported the presence of similar low concentrations of antibiotics (erythromycin, mean 0.35 ppm; penicillin G, mean 0.11 ppm) in 12.6% of 159 distillers grain samples collected from 9 states and 43 ethanol plants in the U.S.^32^. Using various microbiological assays, several studies examined the antimicrobial effects of distillers grains and reported mixed findings^32–34^. In the survey mentioned above, one sample extract (out of 159) inhibited the growth of *E*. *coli* at 10^4^ CFU/ml; however, this sample contained no detectable concentrations of antibiotic residues^32^. A 2015 short communication looking at only one source of commercial dried distillers grains with solubles observed no antimicrobial effect^34^. There are studies reporting the enzymatic degradation of penicillin and erythromycin and their poor stability in bioethanol fermentations^1, 35^; however, a very recent study demonstrated that biologically active virginiamycin at low concentrations (0.69 and 8.9 ppm) persisted in distillers grains produced from fermentations treated with virginiamycin^33^. Several other studies provided evidence that the use of antibiotics such as erythromycin, penicillin, and virginiamycin in ethanol production provides selective pressure for the development of resistant bacteria in the fermentators^36–38^. To our knowledge, there have been three *in vivo* studies to date evaluating the effect of feeding cattle distillers grains containing antibiotic residues on bacterial resistance development, and all reported minimal effects^39–41^. Notably, *Enterococcus* isolates from cattle fed monensin or monensin plus tylosin had greater levels of resistance toward macrolides and there was a tendency for a greater proportion of Q/D-resistant *Enterococcus* in cattle fed distillers grains^39^. Limitations noted include low statistical power and lack of baseline susceptibility data before feeding distillers grains^39–41^.

In conclusion, our *in vitro* study demonstrates that bacterial resistance evolution at sub-MIC concentrations of antibiotics involves a complex interplay between the specific drug, bacterial genetics, and culturing conditions^42, 43^. Further genome-wide identification of resistance determinants among mutants obtained in this study may shed some light on the dynamic mechanisms involved in the process. Although not yet tested *in vivo*, findings from this study suggest the potential risk of stimulating bacterial resistance development when distillers grains containing certain antibiotic residues are used in animal feed. Considering the mixed findings in the literature on this topic and the degradation of antibiotics following fermentation and distillation process, future *in vivo* studies are warranted in order to evaluate such effects in specific food-producing animal species fed commercially produced distillers grains.

## Methods

### Bacterial strains and growth conditions

Ten *Campylobacter* and fifteen *Enterococcus* strains were used as parent strains for mutant selection (Table 1). The strains were recovered from retail meats (ground beef, chicken breast, pork chop, and ground turkey) between 2006 and 2012 by the National Antimicrobial Resistance Monitoring System (NARMS)^44^. Among them, 10 strains from each genus representing five species (*C*. *coli*, *C*. *jejuni*, *E*. *faecalis*, *E*. *faecium*, and *E*. *hirae*) were tested against erythromycin, 10 strains of *Enterococcus* were tested against penicillin, and 5 strains of *E*. *faecium* were tested against virginiamycin (a mixture of two components, virginiamycin M_1_ and virginiamycin S_1_). *Campylobacter* was not tested against penicillin or virginiamycin because neither antibiotic is effective against *Campylobacter* ^45^. Only *E*. *faecium* strains were used in virginiamycin experiments due to the intrinsic resistance of *E*. *faecalis* to streptogramin antibiotics such as quinupristin/dalfopristin (Q/D) and virginiamycin^46^.

*Campylobacter* strains were cultured on blood agar prepared in-house using trypticase soy agar (BD Diagnostic Systems, Sparks, MD) supplemented with 5% horse blood (Remel Products, Lenexa, KS) or in Mueller-Hinton (MH) broth (BD Diagnostic Systems) at 42 °C under microaerophilic conditions (85% N_2_, 10% CO_2_, and 5% O_2_). When organisms were grown in MH broth, 25-cm^2^ tissue culture flasks with vented caps (Corning Inc., Corning, NY) were used. *Enterococcus* strains were grown on MH agar (BD Diagnostic Systems) or in broth at 37 °C.

### Antibiotics and antibiotic-containing media preparation

Erythromycin, penicillin G potassium, and virginiamycin M_1_ and S_1_ were obtained from Sigma-Aldrich (St. Louis, MO). Virginiamycin complex (75% M_1_, 20% S_1_, and 5% other minor analogs) was procured from Santa Cruz Biotechnology, Inc. (Dallas, TX). Antibiotic stock solutions were prepared and stored following guidelines of the Clinical and Laboratory Standards Institute (CLSI)^47^, and aliquots were added to MH broth to obtain three low concentrations (0.1, 0.25, and 0.5 µg/ml) used for mutant selection. Virginiamycin M_1_ and S_1_ were mixed at two ratios (0.5:1 and 5:1) in broths. Aliquots were also added to agar plates (blood agar for *Campylobacter* and MH agar for *Enterococcus*) at or near clinical resistant breakpoint concentrations (see the section below) to screen for resistant mutants at each culture passage. The virginiamycin complex was used to make virginiamycin-containing MH agar plates.

### Mutant selection experiments

**Figure 1:**
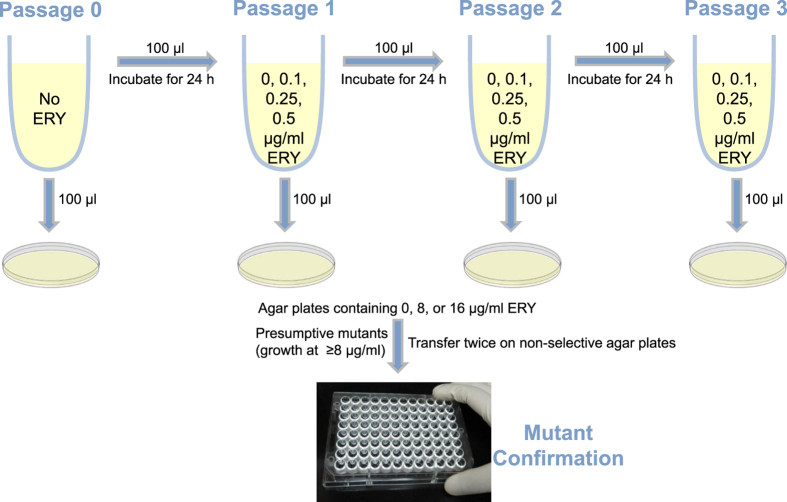
A schematic diagram showing the experimental design used in the erythromycin (ERY) selection experiment.

The procedure used in the FDA/CVM’s preliminary study^11^ was adopted with some modifications. All experiments were independently repeated twice (three times for penicillin). A schematic diagram for erythromycin selection experiments is shown (Fig. 1). Briefly, *Campylobacter* (tested for erythromycin only) and *Enterococcus* (for all three antibiotics) parent strains (Table 1) were cultured overnight in 5 ml of MH broth without antibiotics. Aliquots (100 µl) of the overnight cultures (*ca*. 10^9^ CFU/ml) were transferred to fresh 5 ml of MH broth containing 0, 0.1, 0.25, or 0.5 µg/ml erythromycin, penicillin, or virginiamycin (at M_1_:S_1_ ratios of 0.5:1 and 5:1). After 24 h incubation (approximately 5–6 generations), 100 µl of the cultures were transferred again to fresh MH broth containing the same concentrations and ratios (in the case of virginiamycin) of antibiotics and incubated for 24 h. A total of 3 culture passages were performed for erythromycin, 10–15 for penicillin, and 10 for virginiamycin. For penicillin, strains that did not develop resistance after 10 passages were subject to an additional 5 passages. At each passage, excluding passages 5, 6, 12, and 13 which fell on weekends, the cultures were examined for resistance development by spreading 100-µl aliquots on agar plates (blood agar for *Campylobacter* and MH agar for *Enterococcus*) containing 8 and 16 µg/ml erythromycin, 16 and 32 µg/ml penicillin, or 4 µg/ml virginiamycin complex. Cultures were also plated on respective agar plates without antibiotics. Colonies were enumerated after 24-h incubation for *Enterococcus* and 48-h incubation for *Campylobacter*. Presumptive mutants (2 colonies per selective plate) were subcultured twice on blood agar and stored at −80 °C in brucella broth (BD Diagnostic Systems) containing 20% glycerol.

### *In vitro* antimicrobial susceptibility testing

The minimal inhibitory concentration (MIC) for a panel of antimicrobial agents was determined for each parent and all presumptive mutants. The MICs were determined by broth microdilution using Sensititre NARMS Gram-positive (CMV3AGPF) and *Campylobacter* (CAMPY) MIC plates (TREK Diagnostic Systems, Oakwood Village, OH) for *Enterococcus* and *Campylobacter*, respectively^44^. Q/D was tested as a proxy for virginiamycin. Confirmed erythromycin-resistant mutants were further tested for erythromycin MICs using agar dilution because many had MIC values outside of the test ranges for erythromycin on the Sensititre MIC plates. *Campylobacter jejuni* ATCC 33560 and *Enterococcus faecalis* ATCC 29212 were used as quality control organisms. Susceptibility testing and data interpretation followed CLSI guidelines^47, 48^. Specifically, resistant breakpoints for erythromycin were ≥32 µg/ml and ≥8 µg/ml, respectively, for *Campylobacter* spp. and *Enterococcus* spp. For penicillin, a resistant breakpoint of ≥16 µg/ml was used and for quinupristin/dalfopristin, which was tested as a proxy for virginiamycin, a resistant breakpoint of ≥4 µg/ml was used.

### Statistical analysis

Data on the frequencies of bacterial resistance development were sorted by antibiotic used for selection, bacterial genus, species, strain, antibiotic selection concentration, selection ratio (defined as the ratio between antibiotic selection concentration and parent strain MIC, i.e., MIC_susc._), and number of passages. These data were compared using Chi-square and analysis of variance (ANOVA) (SAS for Windows, version 9.4; SAS Institute Inc., Cary, NC). Differences between the mean values were considered significant when *P* < 0.05.

### Data availability statement

All data generated or analyzed during this study are included in this published article.

## References

[1] U.S. Grains Council. *A guide to distiller’s dried grains with solubles* (*DDGS*) *– 3*^rd^*Edition*, http://www.grains.org/sites/default/files/ddgs-handbook/Complete%202012%20DDGS%20Handbook.pdf (2012).

[2] Renewable Fuels Association. *Building partnerships growing markets*: *2017 ethanol industry outlook*, http://www.ethanolrfa.org/wp-content/uploads/2017/02/Ethanol-Industry-Outlook-2017.pdf (2017).

[3] Olmstead, J. *Bugs in the system*, https://www.iatp.org/sites/default/files/2012_05_02_AntibioticsInEthanol_JO_0.pdf (2012).

[4] Luther, M. *Report of FY 2008 nationwide survey of distillers products for antibiotic residues*, http://www.fda.gov/AnimalVeterinary/Products/AnimalFoodFeeds/Contaminants/ucm331189.htm (2012).

[5] Luther, M. *Report of FY 2010 nationwide survey of distillers products for antibiotic residues*, http://www.fda.gov/AnimalVeterinary/Products/AnimalFoodFeeds/Contaminants/ucm300126.htm (2012).

[6] WHO. *Antimicrobial resistance*: *global report on surveillance*, *2014*, http://apps.who.int/iris/bitstream/10665/112642/1/9789241564748_eng.pdf (2014).

[7] CDC. *Antibiotic resistance threats in the United States*, *2013*, https://www.cdc.gov/drugresistance/pdf/ar-threats-2013-508.pdf (2013).

[8] The White House. *National action plan for combating antibiotic*-*resistant bacteria*, *2015*, https://obamawhitehouse.archives.gov/sites/default/files/docs/national_action_plan_for_combating_antibotic-resistant_bacteria.pdf (2015).

[9] Zhao, X. & Drlica, K. Restricting the selection of antibiotic-resistant mutant bacteria: measurement and potential use of the mutant selection window. *J Infect Dis* **185**, 561–565, doi:10.1086/338571 (2002). 10.1086/338571 11865411

[10] Drlica, K. & Zhao, X. Mutant selection window hypothesis updated. *Clin Infect Dis* **44**, 681–688, doi: 10.1086/511642 (2007). 10.1086/511642 17278059

[11] Blickenstaff, K. *et al*. In 34^th^*Symposium on Biotechnology for Fuels and Chemicals* (New Orleans, LA, 2012).

[12] Scallan, E. *et al*. Foodborne illness acquired in the United States-major pathogens. *Emerg Infect Dis* **17**, 7–15, doi:10.3201/eid1701.P11101(2011). 10.3201/eid1701.P11101 PMC3375761 21192848

[13] Lebreton, F., Willems, R. J. L. & Gilmore, M. S. *Enterococcus* diversity, origins in nature, and gut colonization. In *Enterococci: From Commensals to Leading Causes of Drug Resistant Infection* (eds M. S. Gilmore, D. B. Clewell, Y. Ike, & N. Shankar) (Massachusetts Eye and Ear Infirmary, Boston, MA, 2014). 24649513

[14] FDA. *Guidance for industry #152*: *Evaluating the safety of antimicrobial new animal drugs with regard to their microbiological effects on bacteria of human health concern*, http://www.fda.gov/downloads/AnimalVeterinary/GuidanceComplianceEnforcement/GuidanceforIndustry/ucm052519.pdf (2003).

[15] Andersson DI, Hughes D (2014). Microbiological effects of sublethal levels of antibiotics. Nat Rev Microbiol.

[16] Gullberg, E., Albrecht, L. M., Karlsson, C., Sandegren, L. & Andersson, D. I. Selection of a multidrug resistance plasmid by sublethal levels of antibiotics and heavy metals. *mBio* **5**, e01918–01914, doi:10.1128/mBio.01918-14 (2014). 10.1128/mBio.01918-14 PMC4196238 25293762

[17] Gullberg E (2011). Selection of resistant bacteria at very low antibiotic concentrations. PLoS Pathog.

[18] Liu A (2011). Selective advantage of resistant strains at trace levels of antibiotics: a simple and ultrasensitive color test for detection of antibiotics and genotoxic agents. Antimicrob Agents Chemother.

[19] van der Horst MA, Schuurmans JM, Smid MC, Koenders BB, ter Kuile BH (2011). *De novo* acquisition of resistance to three antibiotics by *Escherichia coli*. Microb Drug Resist.

[20] Andersson DI, Hughes D (2012). Evolution of antibiotic resistance at non-lethal drug concentrations. Drug Resist Update.

[21] Belanger, A. E. & Shryock, T. R. Macrolide-resistant *Campylobacter*: the meat of the matter. *J Antimicrob Chemother* **60**, 715–723, doi:10.1093/jac/dkm300 (2007). 10.1093/jac/dkm300 17704515

[22] FDA. *2014 NARMS Intregrated Report*, https://www.fda.gov/AnimalVeterinary/SafetyHealth/AntimicrobialResistance/NationalAntimicrobialResistanceMonitoringSystem/ucm059103.htm (2016).

[23] Kristich, C. J., Rice, L. B. & Arias, C. A. Enterococcal infection—treatment and antibiotic resistance. In *Enterococci*: *From Commensals to Leading Causes of Drug Resistant Infection* (eds M. S. Gilmore, D. B. Clewell, Y. Ike, & N. Shankar) (Massachusetts Eye and Ear Infirmary, Boston, MA, 2014). 24649510

[24] Zhang X (2012). Genome-wide identification of ampicillin resistance determinants in *Enterococcus faecium*. PLoS Genet.

[25] Luangtongkum T (2009). Antibiotic resistance in *Campylobacter*: emergence, transmission and persistence. Future Microbiol.

[26] Wang, Y. *et al*. Emergence of multidrug-resistant *Campylobacter* species isolates with a horizontally acquired rRNA methylase. *Antimicrob Agents Chemother* **58**, 5405–5412, doi:10.1128/AAC.03039-14 (2014). 10.1128/AAC.03039-14 PMC4135855 24982085

[27] Miller WR, Munita JM, Arias CA (2014). Mechanisms of antibiotic resistance in enterococci. Expert Rev Anti Infect Ther.

[28] Novais, C. *et al*. Co-diversification of *Enterococcus faecium* core genomes and PBP5: evidences of *pbp5* horizontal transfer. *Front Microbiol* **7**, doi:10.3389/fmicb.2016.01581 (2016). 10.3389/fmicb.2016.01581 PMC5053079 27766095

[29] Toprak E (2011). Evolutionary paths to antibiotic resistance under dynamically sustained drug selection. Nat Genet.

[30] Sandegren L (2014). Selection of antibiotic resistance at very low antibiotic concentrations. Ups J Med Sci.

[31] FDA. *Guide for industry #209*: *The judicious use of medically important antimicrobial drugs in food*-*producing animals*, https://www.fda.gov/downloads/AnimalVeterinary/GuidanceComplianceEnforcement/GuidanceforIndustry/UCM216936.pdf (2012).

[32] Compart DM (2013). Presence and biological activity of antibiotics used in fuel ethanol and corn co-product production. J Anim Sci.

[33] Bischoff KM, Zhang Y, Rich JO (2016). Fate of virginiamycin through the fuel ethanol production process. World J Microbiol Biotechnol.

[34] Sankarlal, V. M., Testroet, E. D., Beitz, D. C. & Clark, S. *Short communication:* No antimicrobial effects from one source of commercial dried distillers grains with solubles. *J Dairy Sci* **98**, 8554–8559, doi:10.3168/jds.2015-9932 (2015). 10.3168/jds.2015-9932 26454305

[35] Muthaiyan A, Limayem A, Ricke SC (2011). Antimicrobial strategies for limiting bacterial contaminants in fuel bioethanol fermentations. Prog Energ Combust.

[36] Bischoff KM, Skinner-Nemec KA (2007). & Leathers, T. D. Antimicrobial susceptibility of *Lactobacillus* species isolated from commercial ethanol plants. J Ind Microbiol Biotechnol.

[37] Murphree CA, Heist EP, Moe LA (2014). Antibiotic resistance among cultured bacterial isolates from bioethanol fermentation facilities across the United States. Curr Microbiol.

[38] Murphree, C. A., Li, Q., Heist, E. P. & Moe, L. A. A multiple antibiotic-resistant *Enterobacter cloacae* strain isolated from a bioethanol fermentation facility. *Microbes Environ* **29**, 322–325, doi:10.1264/jsme2.ME13162 (2014). 10.1264/jsme2.ME13162 PMC4159044 24941895

[39] Jacob, M. E. *et al*. Effects of feeding wet corn distillers grains with solubles with or without monensin and tylosin on the prevalence and antimicrobial susceptibilities of fecal foodborne pathogenic and commensal bacteria in feedlot cattle. *J Anim Sci* **86**, 1182–1190, doi: 10.2527/jas.2007-0091 (2008). 10.2527/jas.2007-0091 18192558

[40] Edrington TS, Bischoff KM, Loneragan GH, Nisbet DJ (2014). Evaluation of feeding distiller’s grains, containing virginiamycin, on antimicrobial susceptibilities in fecal isolates of *Enterococcus* and *Escherichia coli* and prevalence of resistance genes in cattle. J Anim Sci.

[41] Edrington TS (2010). Influence of wet distiller’s grains on prevalence of *Escherichia coli* O157:H7 and *Salmonella* in feedlot cattle and antimicrobial susceptibility of generic *Escherichia coli* isolates. Foodborne Pathog Dis.

[42] MacLean RC, Hall AR, Perron GG, Buckling A (2010). The population genetics of antibiotic resistance: integrating molecular mechanisms and treatment contexts. Nat Rev Genet.

[43] Chait R, Palmer AC, Yelin I, Kishony R (2016). Pervasive selection for and against antibiotic resistance in inhomogeneous multistress environments. Nat Commun.

[44] FDA/CDC/USDA. *National Antimicrobial Resistance Monitoring System*, http://www.fda.gov/AnimalVeterinary/SafetyHealth/AntimicrobialResistance/NationalAntimicrobialResistanceMonitoringSystem/default.htm (2017).

[45] Lewis, J. S. & Bush, K. Antibacterial agents. In *Manual of Clinical Microbiology*, *Eleventh Edition* Vol. 1 (eds J. H. Jorgensen *et al*.) (ASM Press, Washington, DC, 2015).

[46] Singh KV, Weinstock GM, Murray BE (2002). An *Enterococcus faecalis* ABC homologue (Lsa) is required for the resistance of this species to clindamycin and quinupristin-dalfopristin. Antimicrob Agents Chemother.

[47] CLSI. Methods for Dilution Antimicrobial Susceptibility Tests for Bacteria That Grow Aerobically; Approved Standard-Eighth Edition (M7-A9). (Clinical and Laboratory Standards Institute, Wayne, PA, 2012).

[48] CLSI. Performance Standards for Antimicrobial Susceptibility Testing; Twentieth Informational Supplement (M100-S22). (Clinical and Laboratory Standards Institute, Wayne, PA, 2012).

